# High spatio-temporal-resolution detection of chlorophyll fluorescence dynamics from a single chloroplast with confocal imaging fluorometer

**DOI:** 10.1186/s13007-017-0194-2

**Published:** 2017-05-23

**Authors:** Yi-Chin Tseng, Shi-Wei Chu

**Affiliations:** 1 Department of Physics, National Taiwan University, No. 1, Section 4, Roosevelt Rd, Da’an District, Taipei City, 10617 Taiwan; 2 Molecular Imaging Center, National Taiwan University, No. 81, Changxing Street, Da’an District, Taipei, 10672 Taiwan

**Keywords:** Optical section, 3D microscopy, Kautsky curve, Chlorophyll fluorescence transient

## Abstract

**Background:**

Chlorophyll fluorescence (CF) is a key indicator to study plant physiology or photosynthesis efficiency. Conventionally, CF is characterized by fluorometers, which only allows ensemble measurement through wide-field detection. For imaging fluorometers, the typical spatial and temporal resolutions are on the order of millimeter and second, far from enough to study cellular/sub-cellular CF dynamics. In addition, due to the lack of optical sectioning capability, conventional imaging fluorometers cannot identify CF from a single cell or even a single chloroplast.

**Results and discussion:**

Here we demonstrated a fluorometer based on confocal imaging, that not only provides high contrast images, but also allows CF measurement with spatiotemporal resolution as high as micrometer and millisecond. CF transient (the Kautsky curve) from a single chloroplast is successfully obtained, with both the temporal dynamics and the intensity dependences corresponding well to the ensemble measurement from conventional studies. The significance of confocal imaging fluorometer is to identify the variation among individual chloroplasts, e.g. the temporal position of the P–S–M phases, and the half-life period of P–T decay in the Kautsky curve, that are not possible to analyze with wide-field techniques. A linear relationship is found between excitation intensity and the temporal positions of P–S–M peaks/valleys in the Kautsky curve. Based on the CF transients, the photosynthetic quantum efficiency is derived with spatial resolution down to a single chloroplast. In addition, an interesting 6-order increase in excitation intensity is found between wide-field and confocal fluorometers, whose pixel integration time and optical sectioning may account for this substantial difference.

**Conclusion:**

Confocal imaging fluorometers provide micrometer and millisecond CF characterization, opening up unprecedented possibilities toward detailed spatiotemporal analysis of CF transients and its propagation dynamics, as well as photosynthesis efficiency analysis, on the scale of organelles, in a living plant.

## Background

Chlorophyll fluorescence (CF) has been proven to be one of the most powerful and widely used techniques for plant physiologists [1–7]. Despite of its low quantum efficiency (2–10% of absorbed light [8]), CF detections are meaningful due to its intricate connection with numerous internal processes during photosynthesis, such as reduction of photosystem reaction centers, non-photochemical quenching, etc. [9, 10]. It is well known that the efficiency of photosynthesis can be derived from CF dynamics, thus providing noninvasive, fast and accurate characterization for photosynthesis. CF characterization has been widely adopted to study plant physiology, including stress tolerance, nitrogen balance, carbon fixation efficiency, etc. [11]. It is not too exaggerated to say that nowadays, no investigation about photosynthetic process would be complete without CF analysis.

Conventionally, the tool of choice to study CF is a fluorometer. There are many different fluorometry techniques, such as plant efficiency analyzer (PEA) [12], pulse amplitude modulation (PAM) [13], the pump and probe (P&P) [14, 15] and the fast repetition rate (FRR) [16]. It is interesting to note that these various detection approaches are all based on the same principle, i.e. the Kautsky effect [7], or equivalent, CF transient when moving photosynthetic material from dark adaption to light environment.

Conventional imaging fluorometers (e.g. PAM and P&P fluorometers) are based on wide-field detection, and are routinely adopted to study ensemble of CF transients from a large area of a leaf, significantly limiting its spatiotemporal resolution. For example, to study stress propagation in a plant leaf [17], current imaging fluorometers only provide spatial resolution on the order of millimeter, with temporal resolution on the order of second. To unravel the more detailed propagation dynamics, the required spatial resolution should be at least on single cell or sub-cellular level, while the temporal resolution should be enhanced to millisecond scale.

The concept of introducing fluorescent microscope to study high-resolution CF dynamics has been realized two decades ago [18], but the drawback of the early microscopic fluorometer version is the lack of optical section capability due to its wide-field nature, and thus prevents study of CF transient on a truly single cell or even a single chloroplast level. Confocal microscopy, which is known to provide optical sectioning with exceptionally high axial contrast, has been extensively used for CF imaging with sub-micrometer resolution [19–23]. However, the high-speed time-lapsed imaging capability is less explored in earlier works.

Here we introduce a concept of confocal imaging fluorometer, which is the combination of confocal microscopy and CF transient detection. The technique not only detects CF signals with millisecond temporal resolution, but also attains micrometer spatial resolution in all three dimensions. The CF transient (Kautsky curve) within a single chloroplast is successfully retrieved. With statistical comparison, the CF transients of a group of palisade cells and the ensemble of single chloroplasts are found to be similar to each other, and both correspond well to the result of conventional imaging fluorometers, showing the reliability of our result. Nevertheless, the CF transient of individual chloroplast can be substantially different, manifesting the value of the unusual capability to study plant cell organelles. Furthermore, we found that the shape of transients is highly intensity-dependent, which is also shown in an earlier study [24]. We also found that the short integration time and optical section characteristic of confocal image fluorometer make a significant difference of illumination intensity comparing to that of conventional fluorometers. Given CF transient from a single chloroplast, it is possible to investigate degree of influence from external or internal plant-stress with scale of organelle, and confocal imaging fluorometer has paved the way for this high spatiotemporal resolution CF detection.

## Principle

### Basic concept of confocal imaging fluorometer

**Fig. 1:**
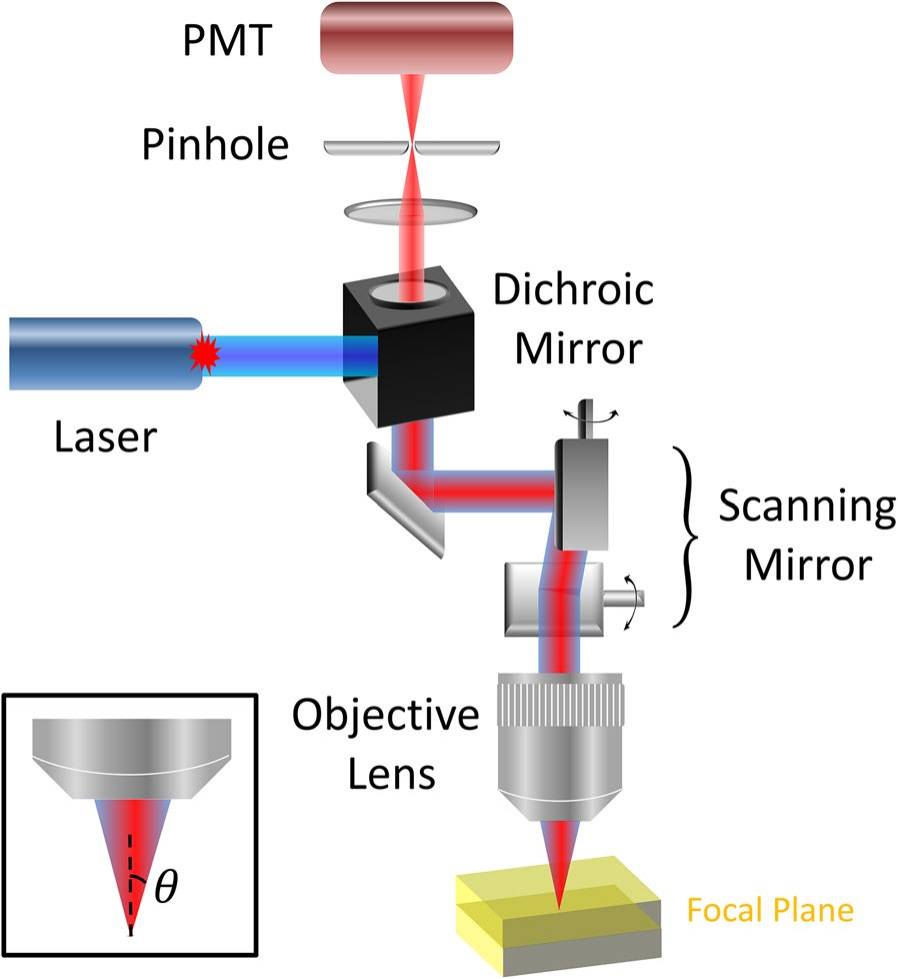
Principle and basic components of a confocal imaging fluorometer. Laser beam is reflected by a dichroic mirror and goes through a set of scanning mirrors, then focused by an objective lens onto the specimen. Fluorescence signals is epi-collected in the same path, and filtered out by the dichroic mirror. A confocal pinhole is used to allow only fluorescence emitted from the focal plane being detected by the PMT

The optical principle of confocal imaging fluorometer is basically the same as confocal laser-scanning microscopy [25], which is an optical imaging technique for increasing contrast and resolution. The essential components of a confocal imaging fluorometer is shown in Fig. 1, including a laser system, a dichroic mirror, a scanning mirror system, an objective lens, a pinhole and a photomultiplier tube (PMT).

The laser system in a confocal imaging fluorometer provides strong and monochromatic illumination, whose wavelength can be selected to meet sample request. The laser beam is sent to the objective after the scanning mirror system to achieve two-dimensional raster scanning at the focal plane. The backward fluorescence signal is collected by the same objective, de-scanned through the scanning mirrors, and separated from residual laser by the dichroic mirror. The fluorescence signal then is focused onto the pinhole, which is placed at the conjugate plane of objective focus, to achieve optical sectioning by excluding out-of-focus signals. One or more PMTs are placed behind the pinhole to collect the in-focus fluorescence signals, which are reconstructed into images by synchronization with the scanning mirrors [25].

In general, a confocal imaging system is capable of collecting signal with a well-defined optical section on the order of 1 µm [26]. This high axial resolution makes confocal system an invaluable tool to observe single cell or sub-cellular organelles [27–29].

The objective lens is characterized by magnification and numerical aperture (NA). To enable large field-of-view observation, low magnification objectives are typically required. However, please note that resolution is determined by NA, which can be independent from magnification. NA describes the light acceptance cone of an objective lens and hence light gathering ability and resolution. The definition of NA is:

1 $$NA \equiv n \times \sin \theta ,$$

where *n* is the index of refraction of the immersion medium, and *θ* is the half-angle of the maximum light acceptance cone. Both lateral (xy-direction) and axial (z-direction) resolutions for fluorescence imaging mode are defined by NA and the wavelength (λ) [30].

2 $$r_{lateral} = \frac{0.43 \times \lambda }{NA}$$

3 $$r_{axial} = \frac{0.67 \times \lambda }{{n - \sqrt {n^{2} - NA^{2} } }}$$

To compare the actinic light illumination in a conventional fluorometer, e.g. PAM, and in a laser scanning confocal fluorometer, there are several aspects. First, in PAM the actinic light is provided by a lamp or an LED, which is an incoherent light source; while in a confocal system, the laser excitation is coherent. Second, the spectral bandwidth of a laser is in general much narrower than that of a lamp, which is typically tens of nanometers even after adding bandpass filters. Third, wide-field illumination is adopted in PAM, while point-scan is used in confocal.

Although there are many differences between the illumination method of the conventional fluorometer and the confocal one, in an early work [31], it has been shown that the actinic effect of using a Xe lamp or a laser is equivalent. In a more recent work [23], they have shown that frequency of scanning (~300 s^−1^) does not seem to affect the response, even when compared to wide-field illumination. In our current work, the scanning frequency on each chloroplast is about 10,000 s^−1^. However, as we will show in the results, clear OPSMT transitions and similar intensity-dependent CF dynamics are all observable. Therefore, it seems that the high-frequency laser beam movement does not cause significant effects on CF dynamics.

### Kautsky effect

Kautsky effect, discovered in 1931, describes the dynamics of CF when dark-adapted photosynthetic chlorophyll suddenly exposes to continuous light illumination [32]. After initial light absorption, chlorophyll becomes excited and soon releases its energy into one of the three internal decay pathways, including photosynthesis (photochemical quenching, qP), heat (non-photochemical quenching, NPQ) and light emission (CF). Owing to energy conservation, the sum of quantum efficiencies for these three pathways should be unity. Therefore, the yield of CF is strongly related to the efficiency of both qP and NPQ [33].

**Fig. 2:**
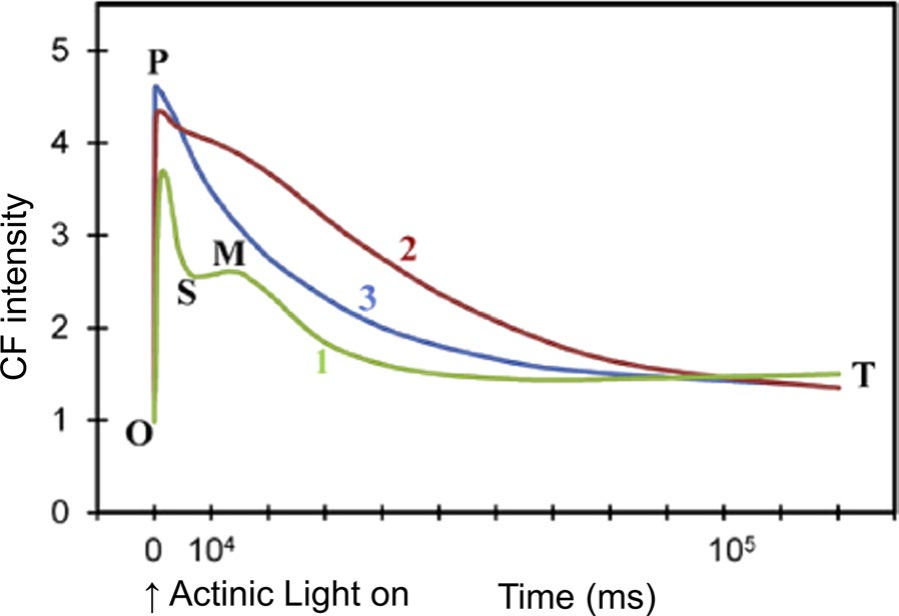
The Kautsky effect, showing the CF transient as well as its intensity dependence. Wavelength of excitation: 650 nm. Excitation light intensity for curves labeled 1, 2 and 3 was 32, 320 and 3200 μmol/m^2^/s, respectively. For definition of OPSMT, O is the origin, P is the peak, S stands for semi-steady state, M for a local maximum, and T for a terminal steady state level (Modified figure from [1], with copyright permission)

To be more specific, when transferring a photosynthetic material from dark adaption into light illumination, CF yield typically exhibits a fast rising phase (within 1 s) and a slow decay phase (few minute duration), as shown by the green curve in Fig. 2. The fast rising phase is labeled as O–P, where O is for origin, and P is the peak [24]. It is mainly caused by the reduction of qP; that is, depletion of electron acceptors, quinine (Qa) in the electron transport chain [34]. The slow decay phase is labeled as P–S–M–T, where S stands for semisteady state, M for a local maximum, and T for a terminal steady state level [24]. One very interesting phenomenon is the shape of this decay phase depends strongly on illumination intensity. At low intensity (32 μmol/m^2^/s), the Kautsky curve is the green one. When the intensity grows one order larger, the amplitude of S–M rise in the transient is smaller, as shown by the red curve. At one more order higher intensity, the blue curve shows that the S–M section disappears completely, leaving an exponential decay in the P–T section. This is known as saturation state, which is critical to derive the quantum efficiency of photosynthesis. Such intensity-dependent curve transition is the result of photosynthetic state transition, and more detailed discussion can be found in the references [1, 10, 13, 35–37].

## Methods

### Plant sample

*Brugmansia suaveolens* (solanaceae), also known as Angel’s Trumpet, was a woody plant usually 3–4 m in height with pendulous flowers and furry leaves distributed widely in Taiwan, especially in wet areas. Being interested in spatiotemporal dynamics of CF, we selected *B. suaveolens* as our target material since the CF of its cousin *Datura wrightii*, also known as Devil’s Trumpet, had been studied in depth [17]. *B. suaveolens* leaves were collected from the Botanical Garden of National Taiwan University, Taipei, Taiwan (25°1′N, 121°31′E, 9 m a.s.l.). All sample leaves were picked as fully expanded leaves that had neither experienced detectable physical damage nor herbivory. In order to minimize the sampling error, three leaves were chosen within plants that grew in similar micro-climate. Furthermore, all the measurements were completed no longer than two hours after disleaving. Fresh leaves were sealed in slide glass (76 × 26 mm), and slide samples were dark-adapted under constant temperature and constant humidity dark environment (20 °C, 70%RH) for 20 min.

### Experimental setup

A confocal microscope (Leica TCS SP5) in the Molecular Imaging Center of National Taiwan University was adopted. CF was excited by a HeNe laser, whose wavelength (633 nm) was the same as that used in popular conventional fluorometers, such as LI-6400 from LI-COR. A relatively low-NA objective (HC PL Apo 10×/0.4 CS) was selected to allow not only large field of view over a few millimeters, but also spatial resolution better than a single chloroplast. From Eqs. (2) and (3), the lateral and axial resolutions were 1 and 5 µm, respectively. Although this was not particularly high compared to common confocal imaging system, due to the low-NA objective here, the three-dimensional spatial resolution was much better than conventional imaging fluorometers.

To operate the confocal fluorometer, the initial step was to bring the sample to focus by weak excitation (~1 kW/cm^2^, or equivalently 5.56 × 10^7^ μmol/m^2^/s for intensity conversion, please see “Discussion”), and then the leaf was left in dark again for 5 min. To observe the Kautsky effect, the 633-nm laser was focused on the sample, and the fluorescence emission was recorded in the spectral range of 670–690 nm. The intensity-dependent CF transient curves were obtained by taking time-lapsed images while varying the 633-nm excitation intensity from 1 to 55 kW/cm^2^, at different sample regions. For experimental details, the scanning speed was 1400 Hz (1400 rows per second), the pinhole size was 52 μm (one Airy diameter), the built-in PMT voltage was set at 600 V, and a dichroic filter TD 488/543/633 was included in the optical path. With different number of total pixels, the temporal resolution of the CF transient varies from 10 ms (16 × 16 pixels) to about 200 ms (256 × 256 pixels). No significant photobleaching of CF was expected at this intensity range [38].

## Results

### Fluorescence dynamics from a single chloroplast

**Fig. 3:**
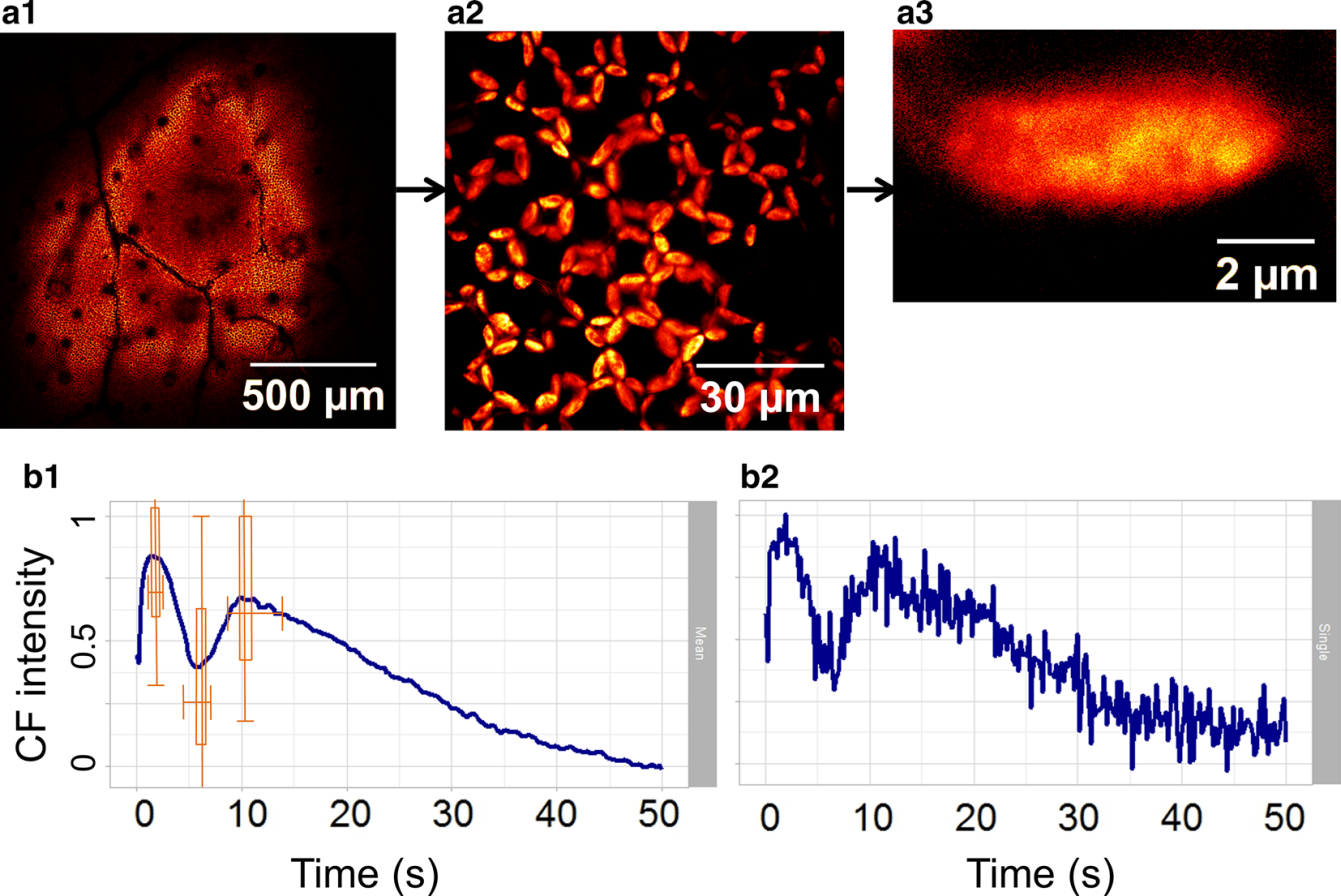
Confocal images and CF transients on different spatial scales inside a living leaf. A 633-nm laser, with 3 kW/cm^2^ intensity, is adopted for 50 s continuous confocal imaging. The sample leaf was kept in darkness for ~20 min before imaging. **a1** The image over a large area of the leaf, **a2** zoomed into show a group of palisade cells, and **a3** further zoomed into focus onto a single chloroplast. **b1**, **b2** CF transient from a group of palisade cells and a single chloroplast, respectively. **b2** Noisier since less pixels are involved

Conventional fluorometers observe CF dynamics over a large area on a leaf, and here we demonstrate that the confocal imaging fluorometer allows us to obtain CF transients from a precisely chosen cell or even a single chloroplast. Figure 3a shows the confocal images of a leaf sample. (a1) is the large-area view, showing the distribution of vascular bundles, while (a2) gives a zoom-in view of a group of palisade cells, showing clear distribution of chloroplasts in each cell. By further zooming in, the field of view is focused onto a single chloroplast, as given in (a3), showing the distribution of chlorophyll density inside the organelle [39].

Figure 3b presents the CF transients at low intensity illumination (3 kW/cm^2^) from a group of palisade cells (b1) and a single chloroplast (b2). The latter is noisier due to less pixels involved. The characteristic P–S decay and S–M rise of Kautsky curve are obvious in both (b1) and (b2). In Fig. 3b1, based on the statistics of 30 chloroplasts, the averaged timing points for P–S–M states are 1.8, 5.9 and 10.4 s, respectively, corresponding well with the reported values in the literature (Fig. 2). On the other hand, box plots are embedded in Fig. 3b1 to show the variations of time and intensity in P–S–M states between the 30 individual chloroplasts. The bottom and top of the box are the first and third quartiles, while the ends of the whiskers represent the maximum and minimum values. This result not only confirms that the averaged Kautsky curves acquired by the confocal fluorometer are similar to the curves taken with conventional fluorometers, but also shows that the variations between individual chloroplasts are indeed significant.

### Intensity dependent fluorescence transient

**Fig. 4:**
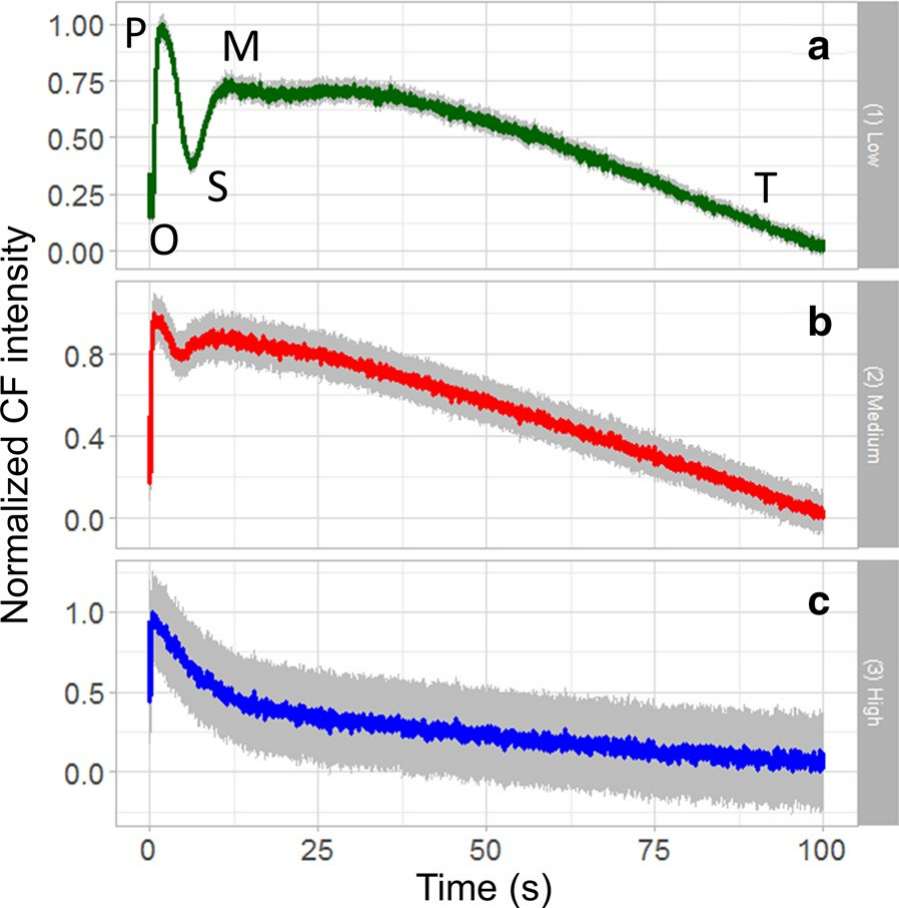
Averaged CF transients from ~780 chloroplasts (*colored*) with standard error (×16, *gray*) under excitation intensity at **a** 3, **b** 10, and **c** 55 kW/cm2, respectively, showing clearly the intensity-dependent Kautsky curves

As we have mentioned in Fig. 2, it is well known that the Kautsky curve changes with intensity. Figure 4 shows the intensity-dependent Kautsky curves from ~780 chloroplasts (colored lines) along with their standard error (gray lines), obtained by the confocal fluorometer. Note that to make the standard error visible on the same scale, it is multiplied by 16. Figure 4a is acquired with low laser intensity (3 kW/cm^2^), and a temporal variation similar to curve 1 of Fig. 2 is found, i.e. a complete O–P–S–M–T curve. The CF intensity rises to its first peak within 1 s (O–P rise), quickly decreasing to a local minimum (P–S fall), rising again to a second peak (S–M rise) then slowly falling as exponential decay (M–T decay). At slightly higher intensity (10 kW/cm^2^), a temporal variation similar to curve 2 of Fig. 2 is observed. The P–S fall and S–M rise still exist, but become much smaller, while the positions of P, S, and M appear earlier in the curve. At high intensity (55 kW/cm^2^), the S–M part disappears completely, leaving a single exponential P–T decay, similar to curve 3 of Fig. 2, i.e. saturation state. This result matches very well to the conventional wide-field fluorometer [1, 13, 36], but with much higher spatiotemporal resolution, manifesting again the reliability and usefulness of the confocal technique.

**Fig. 5:**
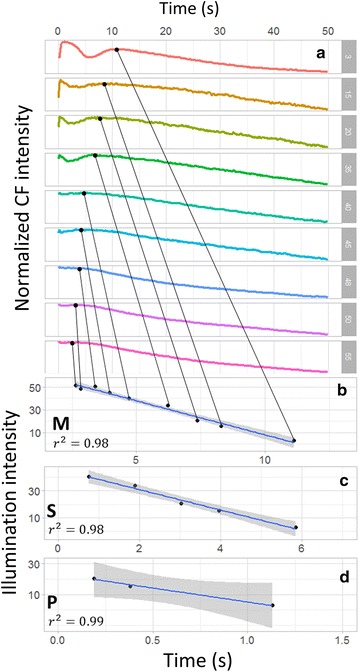
**a** Detailed Kautsky curve variation in the intensity range of 3–55 kW/cm^2^. The temporal positions of **b** the local maximum (M), **c** semisteady state (S), and** d** peak (P), all change linearly with excitation intensity. The *grey area* represents 95% confidence region

From Fig. 4, not only the curve shape is intensity-dependent, but the positions of local maxima and minimum (P, S, M points) are strongly dependent on excitation intensities. Figure 5a shows the detailed curve variation relative to intensity, in the range of 3–55 kW/cm^2^, and the corresponding temporal position of local maximum of induced transients, i.e. point M, is given in Fig. 5b. Surprisingly, an almost perfect linear trend is observed. Similar linear results are found for the semi-steady state point S in Fig. 5c, and for the peak point P in Fig. 5d. Due to the limitation of temporal resolution (200 ms for 256 × 256 pixels), S and P points are analyzed with intensity range 3–40 kW/cm^2^ and 3–20 kW/cm^2^, respectively. The linear trends indicate that the state transition rate increases with higher excitation intensity. The underlying mechanism relies more investigation in the future.

### Fluorescence dynamics under saturation intensity

**Fig. 6:**
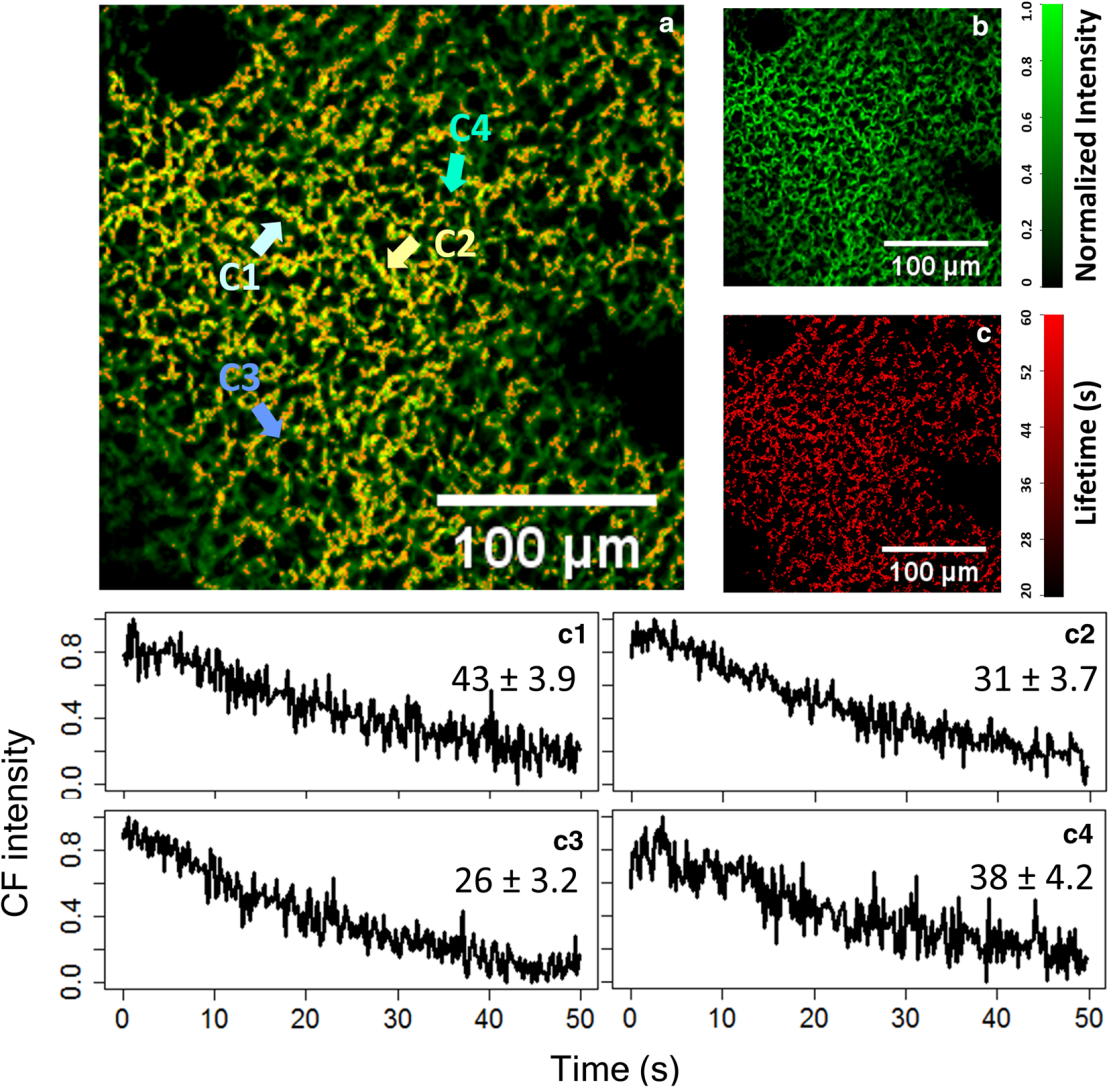
High-resolution spatial distribution of CF intensity (*green* in **a**, **b**) and of the PT-phase time constant (*red* in **a**, **c**). The fluorescence transients of four selected chloroplasts within a living leaf are shown in the *bottom panels*, manifesting the significant difference in the time constants. The dataset is acquired at 40 kW/cm^2^ with a HeNe laser (633 nm)

In the last section, we have shown that at high excitation intensity, the CF is driven into saturation, which is very important for the quantum efficiency calculation. Thus, here we provide further characterization of the saturation states across individual chloroplasts. In Fig. 6a, the green color provides the spatial distribution of CF intensity over many living cells, and the red color shows the distribution of P–T phase decay time constant. For better identification, the two colors are shown separately in Fig. 6b, c. The statistical analysis for the P–T decay time constant of transients from individual chloroplasts is derived from Fig. 6c. The averaged decay time constant of a large area of leaf is 34.6 s, again matching well to the reported values in Fig. 2. Nevertheless, the standard variation of the decay time constant is 10.6 s, which reaches one-third of the average value, so significant divergence exists between each chloroplast. This decay time divergence is manifested by explicitly showing four Kautsky curves from individual chloroplasts in Fig. 6c1–c4.

Please note that error values in Fig. 6c1–c4 are the least square errors when fitting the curves with an exponential decay, different from the statistical standard deviation above. When analyzing data from a single chloroplast, the signal-to-noise ratio is relatively low, resulting in about 10% error in the time constant determination. Fortunately, the variation of time constants among chloroplasts is much larger than 10%, so this error is still tolerable. In the case where reduced error is necessary, the confocal system provides the flexibility to increase the integration time (reducing temporal resolution), so that higher signal-to-noise ratio can be achieved.

**Fig. 7:**
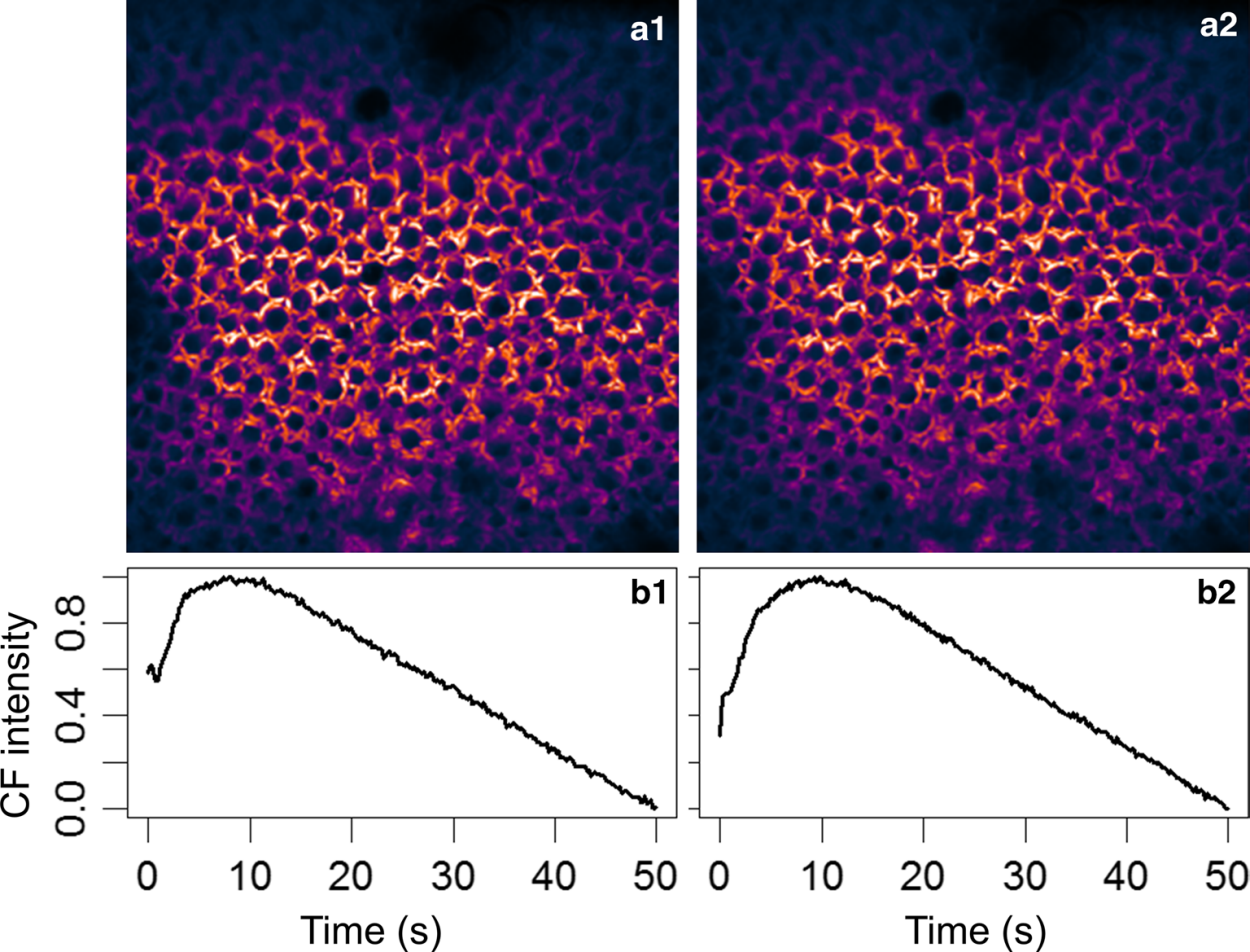
The reproducibility of Kautsky curve under strong illumination intensity (55 kW/cm^2^). **a1**, **b1** are confocal CF image and Kautsky curve for the first set of excitation. **a2**, **b2** are the corresponding results with the second set of excitation after 5 min in dark

Since the laser intensity is relatively strong, it is necessary to confirm the reproducibility of the Kautsky curve in the same region of chloroplasts. Figure 7a1 shows the confocal CF image of a group of cells, and the corresponding averaged Kautsky curve is given in Fig. 7b1. The excitation intensity is 55 kW/cm^2^, which is adequate to saturate the photosystem, so a curve similar to 3 in Fig. 2 is observed. The sample was then kept in dark for 5 min, before the same intensity was applied again. The results of second excitation is given in Fig. 7a2, b2. Apparently, the Kautsky curve is fully recoverable, even under relatively high illumination intensity.

### Deriving quantum efficiency of photosystem II

**Fig. 8:**
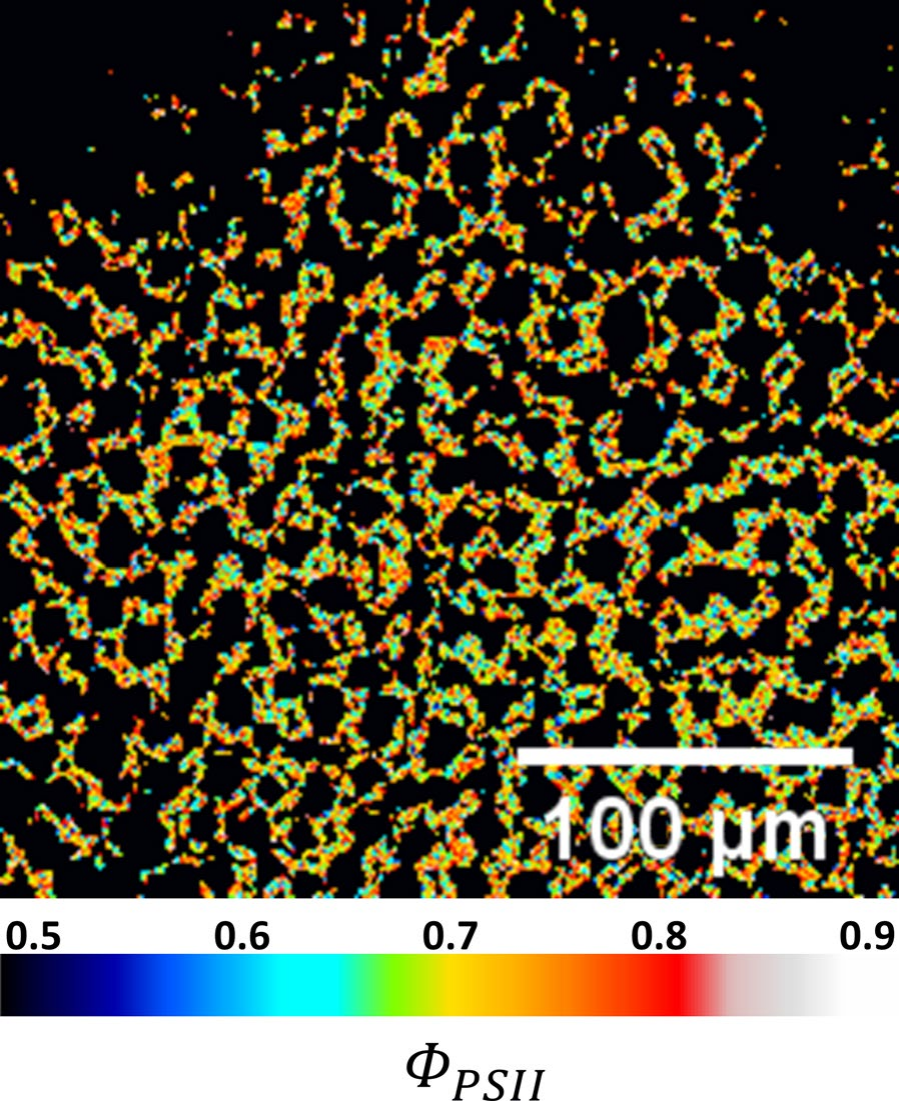
High-resolution spatial distribution of quantum efficiency of photosystem II inside a living leaf

We have shown that intensity-dependent CF transient is found on the scale of cells and chloroplasts, it is then straightforward to derive the physiologically important factors, such as the maximal quantum efficiency of photosystem II (Φ_PSII_). To derive maximal Φ_PSII_, the first step is to quantify the fluorescence yield, which is the ratio between CF intensity and excitation intensity. In our work, the relative quantum yield values are obtained by normalizing the CF intensities to the fluorescence intensity of a commercial fluorescent slide (92001, Chroma Tech., VT) under the same excitation intensity. The values of relative quantum yield at low excitation intensity (Φ_F0_, at 3 kW/cm^2^) and at saturation intensity (Φ_Fm_, at 55 kW/cm^2^) are given in Table 1. Then the spatial distribution of Φ_PSII_ is obtained with pixel-by-pixel calculation of (Φ_Fm_ − Φ_F0_)/Φ_Fm_, as shown in Fig. 8. Apparently, the effect of the fluorescent slide is removed when calculating the quantum efficiency with the above equation. Numerical values of quantum efficiencies on different scales are also listed in Table 1. Similar to the results of Kautsky curves, the mean values of quantum efficiency are similar throughout a large area of leaf to a single chloroplast. On the other hand, from Table 1 and Fig. 8, the value of Φ_PSII_ can be very different among individual chloroplasts, once again manifesting the significance of high-resolution mapping of the CF dynamics inside a living plant.

**Table 1:** Relative quantum yields and quantum efficiencies at different spatial scales

	Large area of leaf (mean/SD)	Group of palisade cells (mean/SD)	Single chloroplast (mean/SD)
Φ_Fm_	47.15/7.76	45.27/6.67	46.77/5.77
Φ_F0_	13.44/3.66	12.46/3.19	11.94/2.44
Φ_PSII_	0.71/0.22	0.72/0.20	0.74/0.16

## Discussion

We have successfully obtained the Kautsky curve, as well as its intensity dependence, with the confocal imaging fluorometer. Comparing to conventional wide-field imaging fluorometers, the confocal technique allows much better spatial confinement due to optical sectioning capability, and thus observation from a single chloroplast becomes possible. With the statistical analyses for P, S, M, T states of the Kautsky curves, at low and high intensities in Figs. 3 and 6 respectively, it can be concluded that the behavior of individual chloroplasts under our confocal imaging fluorometer is indeed similar to a large area of leaf under a conventional wide-field fluorometer. However, the value of the confocal technique lies in the capability to unravel the significant difference between individual chloroplasts, as highlighted by the box plot in Fig. 3 and the clear variation of P–T decay time constants in Fig. 6c.

In terms of the temporal resolution performance, the confocal and wide-field fluorometers should be similar in terms of a single pixel detection, which takes about 1–10 μs in both cases. As mentioned in [17], the wide-field fluorometer takes about 1 s to record one image. Nevertheless, the advantage of the confocal scheme is the freedom to select number of pixels, as well as the position of these pixels, significantly enhancing the temporal responses. By using more advanced scanning approaches, such as random-access microscopy [40], high-speed CF detection among distant chloroplasts is possible. In addition, by adopting a multi-focus scanning approach, such as being demonstrated by spinning disk confocal microscopy in 2009 [23], the frame rate of confocal fluorometer can be significantly improved.

Although spinning disk technique may potentially provide higher frame rate, there are several limitations that prevent it to be an ideal choice for fluorometry application [41]. First of all, due to the size limitation of camera, spinning disk confocal microscopy typically exhibits a small field of view, often only the size of a few cells, which is problematic when studying tissues. A good comparison is given in Fig. 8 of [41], where laser scanning confocal microscope provides much larger field of view.

Second, due to the existence of multiple pinholes on a pinhole array, the optical sectioning capability of spinning disk microscopy is in general less ideal than laser scanning confocal microscopy, especially when observing thick and scattering tissues. In addition, when using a low-magnification lens for large-area study, the spinning disk technique can significantly lose its optical sectioning ability. The reason is that most spinning disk system has a pinhole array comprising pinholes with a fixed size, which is designed for high-magnification and high-NA immersion objective lens, such as a 100×/NA 1.4 objective. However, for plant tissues, low magnification lens with moderate NA is preferred for large area observation. In our case, a 10×/NA 0.4 objective is employed, providing 1.5 mm × 1.5 mm field of view. If the 10× objective is used in a spinning disk system, whose pinhole diameter cannot be adjusted, both the axial sectioning capability and the lateral resolution shall be far less optimal than a confocal system. On the other hand, in a point-scanning confocal system, the pinhole size is easily adjustable, allowing observation for both high and low magnifications. Even with the low NA objective, as we mentioned in the main text, our confocal fluorometer still provides 1-micrometer lateral resolution and 5-micrometer axial resolution, adequate for single chloroplast imaging.

The third concern is image uniformity. In a spinning disk system, when using a Gaussian laser beam, the excitation intensity of the center region is larger than the edge, making it difficult to quantify the response from individual chloroplast. On the other hand, the image uniformity of laser scanning confocal fluorometer is much better than typical spinning disk one.

Last but not least, when comparing spinning disk and laser scanning confocal techniques, it is commonly accepted that the laser intensity at each focus is less for the former, so photobleaching is reduced. However, we would like to point out that the overall accumulated power/energy on the plant tissue is in fact higher, since the laser power is spread over hundreds of foci across the entire field of view. Therefore, more powerful lasers are required for the spinning disk system, and the issue of potential photothermal damage in the tissues has to be considered.

Another important aspect to notice is that the illumination intensity of the confocal fluorometer is much higher than that of the wide-field fluorometers. As shown in Figs. 5 and 6, to eliminate the semi-steady state S in the CF transient, about 55 kW/cm^2^ is required for the confocal fluorometer. However, in the case of wide-field fluorometer, as shown in the example of Fig. 2 [1], to eliminate S, 3200 μmol/m^2^/s is required. Considering the wavelength to be 650 nm in [1], the photon energy is 1240/650 = 1.9 eV = 3 × 10^−19^ J. Therefore, the intensity unit (μmol/m^2^/s) is equivalent to [10^−6^ × 6 × 10^23^ (# of photons)] × [3 × 10^−19^ (J/photon)]/10^4^ cm^2^/s = 18 × 10^−9^ kW/cm^2^. As a result, in the wide-field fluorometer, the required illumination intensity is 3200 × 18 × 10^−9^ kW/cm^2^ = 5.76 × 10^−5^ kW/cm^2^, six orders of magnitude smaller than that in the confocal one.

To explain this 6-order intensity difference, optical sectioning and illumination time of the confocal imaging fluorometer have to be considered. In a conventional fluorometer (wide-field detection), CF signals are emitted throughout the whole leaf in the axial direction, so the depth of field (i.e. signal collection depth) is equivalent to the thickness of a leaf, which is usually 100–1000 µm. On the other hand, for a confocal fluorometer, a pinhole is inserted before the detector to reject most out-of-focus fluorescence, and thus the total signal strength is significantly reduced. The typical depth of field in a confocal fluorometer is about 1–10 µm, which is 2-orders less than that of the wide-field one. Hence, the signal strength of the confocal fluorometer is expected to be 2-orders weaker than the wide-field counterpart.

In terms of the illumination time, in a conventional wide-field imaging fluorometer, the whole leaf sample is illuminated continuously, so the illumination time for each pixel is the same as the frame acquisition time. On the other hand, a small laser focus scans across the sample in the confocal scheme, making the illumination time for each pixel much shorter than the frame time. For example, in the case of Fig. 4a2, 1 frame takes about 1 s, and the frame is composed of 256 × 256 pixels, so the illuminating time for each pixel (1 pixel is roughly 1 µm^2^ in this case) of the confocal imaging fluorometer is about 4-orders shorter than that of conventional wide-field imaging fluorometer.

Combining the above two reasons, it is reasonable that the illumination intensity in the confocal imaging fluorometer needs to be much higher than that in the wide-field fluorometer to achieve similar CF signal strength, as well as the Kautsky curves. The latter is somewhat surprising since it indicates that the physiological response of the chlorophyll remains the same with such high-intensity, yet short-period, illumination. One possible reason is that there is a slow reaction during photosynthesis and CF generation, so the chlorophyll only responses to the average intensity, not the instantaneous intensity. By looking into the electron transport chains in the photosystem, the bottleneck reaction might be the reduction of plastoquinone (PQ), which has a relatively slow reaction rate (100 molChl mmol^−1^ s^−1^) [42]. Further studies are necessary to identify the underlying photochemical mechanism.

## Conclusion

In this work, we demonstrated a confocal imaging fluorometer that can provide high spatiotemporal characterization of CF inside a living leaf. The three-dimensional spatial resolution is on the order of micrometer, and the temporal resolution reaches tens of milliseconds, allowing us to study CF transient, i.e. the Kautsky effect, from even a single chloroplast. Although the ensemble behavior of CF transient, as well as the intensity-dependent Kautsky curves, agree well with the results of conventional wide-field fluorometers, confocal imaging fluorometer provides valuable information toward the difference of CF dynamics among individual chloroplasts. The features of optical sectioning and laser focus scanning in the confocal fluorometer result in much higher illumination intensity compared to conventional techniques, while maintaining normal cellular physiological responses. Our work not only opens up new possibilities to study CF dynamics on the level of organelles, but also is promising to unravel more spatial/temporal details in the associated photosynthetic processes.
